# Septicemia caused by *Eikenella corrodens* in a previously healthy male: A case report

**DOI:** 10.1097/MD.0000000000041849

**Published:** 2025-03-28

**Authors:** Haoting Zhang, Yongfu Xia, Linhong Wang, Bin Zhang

**Affiliations:** a Department of Respiratory and Critical Care Medicine, Binzhou Medical University Hospital, Binzhou, China; b General Medicine Department, Qing Dao Medical University Hospital, Qingdao, China.

**Keywords:** antibiotic, blood culture, case report, *Eikenella corrodens*, septicemia

## Abstract

**Rationale:**

The bacterium *Eikenella corrodens* is part of the normal microbiota of the oropharynx. It typically causes disease in patients with bite wound, immunosuppression, and those undergoing surgery, but rarely affects healthy individuals. Despite the rarity of infection induced by *E corrodens*, early diagnosis and therapy are important.

**Patient concerns:**

The study reports a case of septicemia caused by *E corrodens* in a previously healthy male individual. The patient was admitted to hospital due to fever, fatigue, and mild cough lasting 4 weeks. Physical examination was unremarkable. Chest and abdominal computed tomography showed no obvious infiltrates. Transthoracic echocardiography did not reveal excrescence on the aortic valve.

**Diagnoses:**

*E corrodens* was isolated from blood, proving its pathogenic potential. To confirm the pathogen, 16S rRNA gene sequencing was performed directly from the blood culture, obtaining a sequence that aligned with *E corrodens* with a homology percentage of 99.8%. The patient was diagnosed as septicemia caused by *E corrodens*.

**Interventions:**

The patient was treated with moxifloxacin (0.4 g once daily) intravenously for 3 days, but continued to experience recurrent fever. Following the diagnosis of septicemia caused by *E corrodens*, antibiotic treatment was switched to cefoperazone-sulbactam (SCF; 3 g every 8 h) for 14 days in total.

**Outcomes:**

Fever, fatigue, and other discomfort were relieved after 1 day of treatment with SCF. The white blood cell count, C-reactive protein concentration, and procalcitonin levels were gradually normalized after 14 days of treatment with SCF. Blood culture was performed after 14 days of treatment with SCF, yielding negative results.

**Lessons:**

The present case report highlights that *E corrodens* should be considered as a cause of septicemia even in healthy individuals. For the avoidance of delayed diagnosis, it is vital to perform blood culture before initiating antibiotic treatment. Furthermore, SCF should be considered as a preferred treatment option for such infections.

## 1. Introduction

The bacterium *Eikenella corrodens* (*E corrodens*) is a member of the HACEK group (*Haemophilus* spp., *Aggregatibacter* spp., *Cardiobacterium* spp., *E corrodens*, and *Kingella kingae*) belonging to the Neisseriaceae family. It is a part of the normal mucosal microbiota; although it mainly inhabits the oropharynx, it is also present in the upper respiratory tract, digestive tract, and urinary tract.^[1]^ The species is a slow-growing, fastidious, and facultative anaerobic gram-negative bacterium. It was named after its ability to produce pitting on agar. This bacterium rarely causes disease in previously healthy adults. However, in patients with bite wound or immunosuppression, and those undergoing surgery, it may transfer through the bloodstream and induce infections.^[2,3]^ With the increase in the prevalence of predisposing factors (e.g., bite wound and trauma), infections caused by *E corrodens* are becoming increasingly complex and diverse. Such infections range from superficial skin infection to severe systemic damage, with reported cases of endocarditis, pericarditis, osteomyelitis, etc.^[4–7]^ However, *E corrodens* has caused infections in some healthy individuals without potential risk factor. In those cases, it was difficult to achieve a definitive diagnosis at initial presentation. In this article, we describe a case of septicemia caused by *E corrodens* in a previously healthy male. The objective of this report is to share clinical practice experience regarding the diagnosis and treatment of infections caused by *E corrodens*.

## 2. Case presentation

A 65-year-old Chinese male with an unremarkable medical history presented to Hospital due to fever, fatigue, muscle pain, mild cough lasting 4 weeks, as well as unintended weight loss of 3 kg. He had no history of recent travel, drug abuse, or blood transfusion. Based on the symptoms, the patient was admitted to the Department of Respiratory Diseases. On admission, his blood pressure was 103/69 mm Hg, the heart rate was 78 beats per minute, and his temperature was 38.2 °C; oxygen saturation was 97% without oxygen supplementation. On auscultation, crackles were not detected in either of the lungs. Further clinical examination was unremarkable, and he was not in acute distress at any point during his stay in hospital.

Laboratory examination revealed elevated white blood cell count (11.5 × 10^9^/L), C-reactive protein (55.5 mg/L), procalcitonin (1.357 ng/mL), and erythrocyte sedimentation rate (81 mm/h). Chest and abdominal computed tomography showed no obvious infiltrates. Transthoracic echocardiography did not reveal excrescence on the aortic valve. Several analyses (i.e., pharyngeal swabs, thyroid function, urine routine, *Brucella melitensis* antibody, and T-Spot test) yielded negative results. In addition, the levels of antinuclear antibody and anti-neutrophil cytoplasmic antibody were normal.

**Figure 1.**
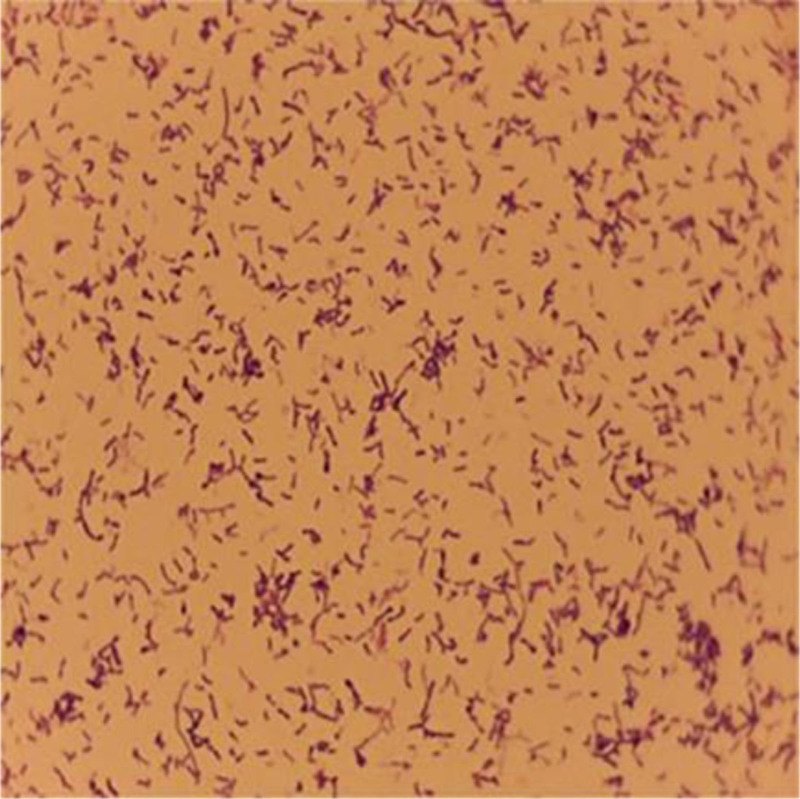
Gram staining of the culture showing gram-negative bacilli.

**Figure 2.**
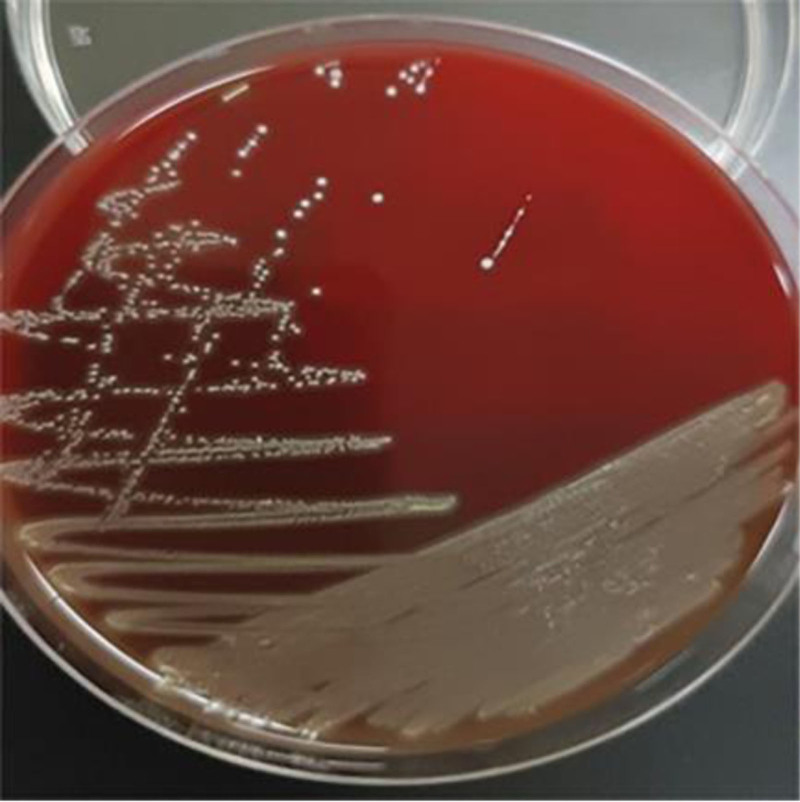
Pale yellow colonies of *Eikenella corrodens* on Columbia agar.

According to the principle and procedure established by the Clinical and Laboratory Standards Institute (CLSI M47-A), blood culture was performed before commencing the antibiotic treatment.^[8]^ Initially, blood was collected from the left and right limb veins at the time of fever. Thereafter, blood (10 mL) was injected into 2 aerobic and 2 anaerobic BACT/ALERT bottles (bioMérieux, France), respectively. Subsequently, all bottles were transported directly to the laboratory by medical staff within 2 hours and were incubated in an automatic BACT/ALERT 3D instrument (bioMérieux, France) at 37 °C. The blood in 2 anaerobic bottles proved positive after about 60 and 62 hours, while 2 aerobic bottles yielded positive results after about 66 and 68 hours. Gram staining showed that gram-negative bacilli (Fig. 1) were subcultured on Columbia agar (Sigma–Aldrich, USA) in aerobiosis with 5% of CO_2_ at 37 °C. After 72 hours, a subtle growth of pale yellow colonies on Columbia agar was observed (Fig. 2). The colonies were identified as *E corrodens* using a VITEK MS (bioMérieux, France) according to the instructions provided by the manufacturer. To confirm the pathogen, 16S rRNA gene sequencing was performed directly from the blood culture, obtaining a sequence that aligned with *E corrodens* with a homology percentage of 99.8%. The sequence was registered in GenBank (accession number: OR501905). Antimicrobial susceptibility testing of the strain was performed on fastidious Muller–Hinton agar (Oxoid, UK) according to CLSI M45-A3 document,^[9]^ and the bacterium was susceptible to ceftriaxone, cefotaxime, ampicillin-sulbactam, amoxicillin-clavulanate, imipenem, meropenem and trimethoprim-sulfamethoxazole, but was resistant to azithromycin, clarithromycin and levofloxacin.

In the search for the origin of infection, there were no signs of tooth abscess, periodontitis or oral ulcer; nevertheless, extremely poor dental hygiene status was observed. The patient was treated with moxifloxacin (0.4 g once daily) intravenously for 3 days, but continued to experience recurrent fever. Following the diagnosis of septicemia caused by *E corrodens*, antibiotic treatment was switched to cefoperazone-sulbactam (SCF; 3 g every 8 h) for 14 days in total. Fever, fatigue, and other discomfort were relieved after 1 day of treatment with SCF. The white blood cell count, C-reactive protein concentration, and procalcitonin levels were gradually normalized after 14 days of treatment with SCF. Blood culture was performed after 14 days of treatment with SCF, yielding negative results. At a follow-up performed in an outpatient clinic 1 month after discharge, the patient was in good health status and asymptomatic with normalized biochemistry.

## 3. Discussion

This is a typical case of septicemia caused by *E corrodens* in a previously healthy individual. The report highlights that *E corrodens* should be considered as a pathogenic factor of septicemia. Although septicemia is common, septicemia caused by *E corrodens* is relatively rare. Previous or concomitant illness has been strongly associated with *E corrodens* infections. According to the literature, most patients with *E corrodens* infections had clear potential predisposing factors, such as diabetes, tumor, and surgery.^[1]^ These risk factors may be responsible for serious *E corrodens* infections.

In recent years, infections caused by *E corrodens* have been increasingly reported in previously healthy individuals. In the present case, although the patient did not have any predisposing factors, he had poor oral hygiene. Septicemia induced by oral uncleanness have been reported in several cases.^[10,11]^ *E corrodens* is a colonizer of the human oral cavity and present in periodontally healthy individuals. Therefore, poor oral hygiene may cause mucosal damage, promoting the entry of bacteria to the bloodstream and inducing septicemia.

Patients with septicemia often present with fever, shivers, shortness of breath, tachycardia, rash, joint swelling, and pain. In this case, the patient had a protracted disease course with fever, fatigue, and mild cough, as well as unintentional weight loss of 3 kg. Moreover, the diagnosis of septicemia was not reached until blood culture was performed. *E corrodens* is an opportunistic pathogen inhabiting the oropharynx, and may cause extraoral infection by blood transmission in patients with oral trauma and those undergoing surgery. Infections induced by *E corrodens* may lead to abscesses in various organs and tissues, such as the brain,^[12]^ thyroid,^[13]^ lungs,^[14]^ liver,^[4]^ pancreas,^[15]^ kidneys,^[16]^ vulvar abscess,^[17]^ intramuscular abscess^[18]^; the strain was isolated from the pus in the cultures. In this case, the previously healthy individual did not develop obvious positive symptoms and signs, except for the symptoms of septicemia (e.g., fever, fatigue, and myalgia). Thus, in clinical practice, *E corrodens* should be considered as one of the pathogenic bacteria that may induce septicemia.

In the absence of risk factors, *E corrodens* is typically overlooked by doctors, thereby aggravating the condition of the patient. Therefore, it is vital to obtain microbiological samples before initiating antibiotic treatment. *E corrodens* may often be disregarded in gram staining. Therefore, it is important to conduct aerobic and anaerobic cultures, or the examination of biological specimens collected from infections which may be caused by anaerobic or facultative anaerobic bacteria. It is likely that *E corrodens* may be missed in blood culture due to its slow growth in the absence of CO_2_ supplementation or misidentified due to its morphological resemblance to other organisms, such as *Haemophilus* spp. When blood cultures yield negative findings, lesions should be identified and biopsied for culture and metagenomic next-generation sequencing, which offer high sensitivity for early diagnosis.^[3]^ In addition, *E corrodens* infections are described as part of polymicrobial infections.^[14,19]^ In this case, there were no other microorganisms isolated from blood. Moreover, traditional laboratory examinations, ultrasound examinations, and imaging examinations did not detect tissue and organ infections.

The selection of antibiotics is particularly important for the treatment of infection induced by *E corrodens*. According to the literature, *E corrodens* is resistant to treatment with macrolides, aminoglycosides, and antimicrobials traditionally used against anaerobes, such as clindamycin and metronidazole. Hence, it is recommended that penicillins and third-generation cephalosporins or tetracyclines are used for the treatment of *E corrodens* infections. Moreover, previous studies described large variations between the different generations of cephalosporins. Specifically, third-generation agents were markedly more potent against *E corrodens* infections than first-generation agents.^[20]^ It has been reported that appropriate and sufficient antibacterial treatment is important for a good outcome.^[21]^ In the present case, the patient was initially treated with intravenous moxifloxacin (0.4 g once daily) for 3 days; nevertheless, his fever persisted. When blood cultures yield positive for *E corrodens*, antimicrobial susceptibility testing of the strain was performed according to CLSI M45-A3 document. The bacterium was susceptible to third- or fourth-generation cephalosporins, penicillin/β-lactamase inhibitor and carbapenems. The patient received 14 days of intravenous treatment with SCF (3 g per 8 h) to cover anaerobic pathogens, as previously suggested in other case report.^[6]^ Finally, the clinical symptoms improved significantly. The observed improvement in this patient after reintroduction of SCF indicates that monotherapy with moxifloxacin may be insufficient; thus, SCF might be a better choice in this setting.

## 4. Conclusion

Although *E corrodens* is a commensal bacterium of the oropharynx in humans, it may cause severe infections when transferred to other anatomical sites. Despite its limited pathogenicity, *E corrodens* should be considered as a potential causative agent in cases of septicemia. Therefore, it is crucial to correctly identify this microorganism and understand its role in infections to achieve prompt and proper treatment.

## Acknowledgments

This work was supported by University-Industry Collaborative Education Program (No. 230805236083924), and Shandong Province Medical and Health Science and Technology Project (202401060833).

## Author contributions

**Investigation:** Haoting Zhang, Yongfu Xia.

**Software:** Haoting Zhang, Yongfu Xia.

**Writing – original draft:** Haoting Zhang, Yongfu Xia, Bin Zhang.

**Writing – review & editing:** Haoting Zhang, Linhong Wang, Yongfu Xia, Bin Zhang.
